# SAFS: Object Tracking Algorithm Based on Self-Adaptive Feature Selection

**DOI:** 10.3390/s21124030

**Published:** 2021-06-11

**Authors:** Wenhua Guo, Jiabao Gao, Yanbin Tian, Fan Yu, Zuren Feng

**Affiliations:** 1 State Key Laboratory for Manufacturing Systems Engineering, Xi’an Jiaotong University, Xi’an 710049, China; markguo@xjtu.edu.cn (W.G.); fzr9910@xjtu.edu.cn (Z.F.); 2 School of Computer Science and Technology, Xi’an Jiaotong University, Xi’an 710049, China; tianyanbin@stu.xjtu.edu.cn (Y.T.); fanyu.cs@stu.xjtu.edu.cn (F.Y.)

**Keywords:** object tracking, self-adaptive feature selection, feature sub-template, maximum a posteriori

## Abstract

Object tracking is one of the most challenging problems in the field of computer vision. In challenging object tracking scenarios such as illumination variation, occlusion, motion blur and fast motion, existing algorithms can present decreased performances. To make better use of the various features of the image, we propose an object tracking method based on the self-adaptive feature selection (SAFS) algorithm, which can select the most distinguishable feature sub-template to guide the tracking task. The similarity of each feature sub-template can be calculated by the histogram of the features. Then, the distinguishability of the feature sub-template can be measured by their similarity matrix based on the maximum a posteriori (MAP). The selection task of the feature sub-template is transformed into the classification task between feature vectors by the above process and adopt modified Jeffreys’ entropy as the discriminant metric for classification, which can complete the update of the sub-template. Experiments with the eight video sequences in the Visual Tracker Benchmark dataset evaluate the comprehensive performance of SAFS and compare them with five baselines. Experimental results demonstrate that SAFS can overcome the difficulties caused by scene changes and achieve robust object tracking.

## 1. Introduction

With the rapid development of hardware facilities and artificial intelligence technology, object tracking has become more important within the domain of computer vision. Object tracking has many applications in real life, such as video surveillance [1], athlete competition analysis, and intelligent human–computer interaction [2].

Traditional tracking algorithms can be divided into two types based on their appearance models: generative and discriminative trackers. Using the object’s appearance information, generative trackers create an appearance model and search for the most similar candidate in each frame with the least amount of reconstruction error. Ross [3] demonstrates a monitoring approach that discovers a low-dimensional subspace representation incrementally and digitally updates it to respond to changes in the object’s presence. In [4,5], the model of tracking is a sparse approximation problem in which a number of trivial templates are added to deal with difficult problems such as particle occlusion, scale variance, etc. Riahi D et al. [6] propose a novel generative approach for multiple object tracking using a multiple feature framework. Tkach A et al. [7] design a real-time generative object tracking based on an online optimization algorithm that jointly estimates pose and shape in each frame, and determines the uncertainty in such estimates. On the other hand, discriminative trackers locate the object through the learning of a decision that limits the object and the context. Helmut Grabner et al. [8] suggest an approach to pick features for monitoring objects for online boosting. In the CVPR 2013 tracking benchmark [9], Sam Hare et al. [10] use an online standardized output support vector machine (SVM) to map objects. Kalal et al. [11] add a tracking re-detection module, which can reboot tracking if the object is totally overstepped or does not appear in the scene. The feature selection is a critical task in object tracking, so many researchers are trying to improve the self-adaptability of this module to overcome this challenge. Different correlation filters have recently been proposed to help traditional object tracking algorithms achieve robust performance and better results [12,13,14]. Furthermore, Zhang J et al. [15] suggest a robust target detection, Multi-task Correlation Particle Filter, for which the strengths of multi-task correlation filter (MCF) and a particle filter are used and improved. Perez-Cham O. E. et al. [16] apply the bio-inspired algorithms to object tracking, which is based on the parallelization of the Honeybee Search Algorithm, and employs the Zero Mean Normalized Cross-Correlation criterion as a fitness function. In addition, after considering the frequent occlusion of target objects in the complex scene, Bae S H et al. [17] propose a robust online multi-object tracking (MOT) approach. Chen Y et al. [18] design a novel Adaptive Focused Discriminative (AFOD) segmentation tracker with the following advanced components. Varfolomieiev A [19] proposes the improvements for visual object trackers based on discriminative correlation filters using channel-independent spatially regularized method for filter calculation. Unfortunately, although these strategies can solve this problem, they are often based on deep learning, which needs large-scale labeled data and computing resources. Tschannen M et al. [20] propose a general framework for self-supervised learning of transferable visual representations based on Video-Induced Visual Invariances (VIVI). Although some workers have been proposed to overcome those challenges, such as self-supervised, transfer learning or semi-supervised learning. However, those methods still require a deep neural network. The ever-increasing requirements of those models may raise the threshold for training them on the edge devices.

In this paper, we purpose SAFS, an object tracking method based on a self-adaptive feature selection algorithm. Our method selects the suitable tracking feature by one new strategy. In this manner, we compare the similarity of each feature sub-template, and then measure the separability of different features of the object using the maximum a posteriori (MAP). Lastly, we adopt modified Jeffreys’ entropy as the discriminant metric for this task. Through updating the feature sub-pattern, our model can update the target template and reduce the drift of the template. To evaluate the effectiveness of the algorithm, we implement our algorithm using color and texture features and apply it in eight different video sequences. Our proposed model can obtain better success rates and reduce the center location errors. Especially in the complex environment (such as basketball), the SAFS has improved above 90% performance than other baselines. In addition, these results demonstrate that our proposed algorithm can achieve higher robustness and faster inference speed.

The main contributions of our paper can be described as follows:

- We characterize the sub-template of each feature and calculate the similarity value matrix of each sub-template based on the linear property of the maximum posterior probability [21].
- We optimize the calculation of Jeffreys’ entropy [22] and use it as a metric of similarity value, which can measure the difference between categories more efficiently.
- We propose an object tracking method based on a self-adaptive feature selection algorithm, called SAFS, which can obtain better tracking performance without human labor.

## 2. Adaptive Multi-Feature Selection Tracking Algorithm

**Figure 1:**
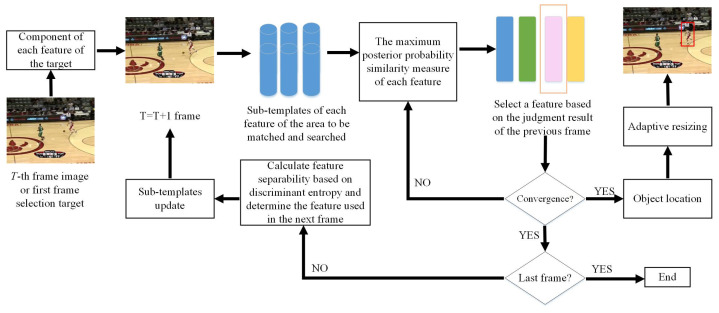
The framework of object tracking algorithm based on self-adaptive feature selection.

Feature selection of object tracking is a hard mission. We use different image features to calculate the similarity between the target area and the area to be matched as the criterion to select the most appropriate tracking feature. The adaptive feature selection mechanism can make full use of the adaptability of different features to different scenarios. When the environment changes, select the most distinguishable feature to track the target stably. This method also has good extensibility, in that it can combine various features in an algorithm to track objects. Based on this idea, we designed the object tracking algorithm based on adaptive feature selection. The framework of the object tracking algorithm adopts the template matching method of generative class. According to the similarity value, it is necessary to measure the ability of each feature to distinguish the foreground and the background, so as to select the appropriate feature in the tracking of the next frame. The framework of the algorithm shows in Figure 1.

The framework mainly includes three steps:

(1) Description of object features: calculate the description of each feature of the target area, the area to be matched, and the searching area, respectively. Including the feature description of the target in the first frame and the feature description of the searching area and the area to be matched in each subsequent frame.
(2) Flexibly select the most proper feature: the optimal feature calculated in the previous frame is selected as the descriptive feature of this frame in the tracking process. Then, the object location is calculated iteratively according to the maximum posterior probability similarity criterion.
(3) Selection for multi-feature: according to the central position of the object, divide the foreground and background of the searching area, and calculate the similarity of each feature. According to the similarity value map, the j-divergence entropy [22] is used to compute the ability of different features to distinguish the foreground and background. Select the most distinguishing feature according to the j-divergence entropy [22], and use this feature in the next frame tracking.

### 2.1. Multi-Feature Description and Similarity Measure of Sub-Template

This algorithm separately calculates the value of each feature operator of the target, constructs the sub-template of each feature of the target, and then combines the sub-templates of each feature to describe the target. In this way, two purposes can be achieved:

1. Decrease the feature vector dimension of multiple features;
2. Flexibly select the most proper feature.

Assume:

The template of target area: ${\left\{q_{u}^{i}\right\}}_{i,\ldots ,n}$
The template of area to be matched: ${\left\{p_{u}^{i}\right\}}_{i,\ldots ,n}$
The template of searching area: ${\left\{s_{u}^{i}\right\}}_{i,\ldots ,n}$

In the above formula, *n* represents the feature vector dimension of one sub-template; $q_{u}^{i}$ denotes the sub-template described by the *i*-th feature in target area; $p_{u}^{i}$ denotes the sub-template described by the *i*-th feature in area to be matched; $s_{u}^{i}$ represents the sub-template described by the *i*-th feature in searching area.

The features of each sub-template are described by feature statistical histogram. Using the tracking algorithm based on maximum posterior probability [21] similarity criterion to calculate each sub-template’s similarity contribution value $g^{i}\left(x_{j}\right)$ and similarity function value $\rho_{yj}$:

(1) $$\begin{array}{c} \rho_{yj}^{i}=\sum_{x_{j}\in A_{yj}}g^{i}\left(x_{j}\right)\\ g^{i}\left(x_{j}\right)=\frac{q_{u}^{i}\left(x_{j}\right)}{s_{u}^{i}\left(x_{j}\right)} \end{array}$$

where ${\left\{x_{j}\right\}}_{i=1,\ldots ,m}$ denote positions of each pixel in searching area; $y_{j}$ represents the central point of this area; $g^{i}\left(x_{j}\right)$ denotes the similarity contribution value of each pixel of the *i*-th feature sub-template in searching area; $A_{yj}$ represents the aggregation of pixels centered in searching area of $y_{j}$; $\rho_{yj}$ denotes the similarity function value of the *i*-th sub-template in searching area.

Based on the sub-templates described by each feature of target area, area to be matched and searching area, we can figure out the feature map corresponding to the similarity contribution value of each sub-template. According to the linear property of the maximum posterior probability [21], the similarity value matrix of each sub-template can be calculated.

Different sub-templates have different similarity value matrices. Some features have a stronger distinguishing ability, so their similarity value matrix has a higher peak in the target area and the value in the edge part far away from the target in the searching area is lower. The closer the matrix is to the target center, the higher the similarity value. Some features are weak in distinguishing ability, so the values of the edge parts away from target have only a small difference between the target area and searching area of their similarity value matrix. Once we design one criterion to measure the difference between target position and edge part of searching area in the similarity value matrix of each sub-template, we can describe the distinguishing ability of sub-template in this frame, and use it to instruct the tracking of the next frame.

### 2.2. Description of Feature Selection

Obtaining the prior information of the scene is a hard mission; even if the prior information of the scene can be obtained by the machine learning method, the information is still uncertain as the environment changes. Therefore, if the tracking algorithm relies on a feature for long-term continuous tracking, it will lose the object easily. One way to solve this problem is to determine the distinguishing ability of each feature of target in current scene in real-time. The discriminant tracking algorithm is to deal with the tracking task along this way: regard tracking as a two-category task of foreground and background, then design a classifier and find the optimal classification result to obtain the object. We can design a new tracking algorithm based on this idea. Assuming that a feature takes into account both the accuracy of the feature’s description and the separability of foreground and background, this feature will show good tracking results during the object tracking process. Therefore, we can determine the tracking features of the current frame by designing an algorithm to compute the separability of different features of the object in the scene.

By sampling the tracked foreground and background separately, and then using mathematical tools to calculate the distance between the two classes, the distinguishability of different features can be measured. The sampling process can uniformly sample *M* regions with the same target size as positive examples in the target center according to the principle of the ordinal optimization, and uniformly sample *N* regions with the same target size as negative examples around the background of the target. The bigger the difference is between positive examples and negative examples, the better the distinguishing ability is of the sub-template. If more positive examples and negative examples are chosen, the separability measure of the judging feature will be more accurate. However, if too many examples are chosen, it will bring the problem of increasing computation complexity. In this paper, the maximum posterior probability similarity criterion is used to measure the similarity between the target and the positive example and the similarity between the target and the negative example. Therefore, the linear computation property of the maximum posterior probability can be used to quickly calculate the similarity value of the feature.

Assume the similarity between the target and positive example: $\rho_{m}^{i,pos}$, where *i* denotes the *i*-th sub-template and m denotes the similarity of the *m*-th positive example.

Assume the similarity between the target and negative example: $\rho_{n}^{i,neg}$, where *i* represents the *i*-th sub-template, *n* represents the similarity of the *n*-th negative example.

**Figure 2:**
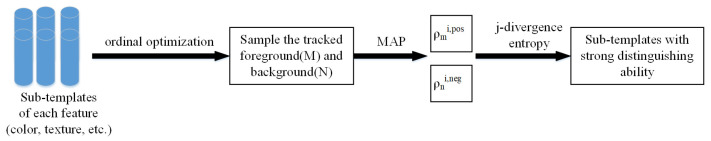
The selection process of feature sub-template.

Now, the selection task of sub-templates is turned into a classification task of two sets of feature vectors: a set of similarity values $\rho_{m}^{i,pos}$ between positive examples and the target, and a set of similarity values $\rho_{n}^{i,neg}$ between negative examples and the target. There are many criteria for discriminating metrics: distance metric, fisher discriminant criteria [23], etc. We select the relative entropy [24] and j-divergence entropy [22] as the discriminating metric of classification. The selection process of feature sub-template of next frame is shown in Figure 2.

### 2.3. Introduction of Relative Entropy and j-Divergence Entropy

Relative entropy [24] is a measure of the asymmetry of the difference between two probability distributions *P* and *Q*. The physical meaning of relative entropy is the metric of the number of extra bits required to encode samples from *P* using *Q*-based coding. Normally, *P* represents the actual distribution of the data, *Q* represents the theoretical distribution of the data, the template distribution, or the approximate distribution of *P*. It has four characteristics:

1. It can be used to measure the similarity between two functions whose values are positive.
2. For two functions that are entirely the same, their relative entropy is zero. The bigger the difference is, the bigger the relative entropy is.
3. If the values of two randomly distributed probability density functions are greater than zero, the relative entropy can measure the difference between the two random distributions.
4. Relative entropy is not symmetric, and commutative law does not work.

We consider two situations in our paper. Assume that two kinds of feature vector are $P={\left\{p_{i}\right\}}_{i=1}^{n}$ and $Q={\left\{q_{i}\right\}}_{i=1}^{n}$, and $\sum p_{i}=\sum q_{i}=1$ is satisfied. The relative entropy D(P,Q) of these two feature vectors is defined as:

(2) $$D\left(P,Q\right)=\sum_{i=1}^{n}p_{i}\log\frac{p_{i}}{q_{i}}$$

where *n* is the dimension of the feature.

Obviously, D(P,Q)≤ 0. D(P,Q) is equal to 0 if and only if *P* equals to *Q*. In other words, the bigger the difference is between these two kinds of random distribution, the bigger the relative entropy is between them. When relative entropy equals zero, they have the same distribution.

From the formula of relative entropy, we can know relative entropy is asymmetric. To solve the asymmetry, the discriminant entropy J(P,Q) (also called Jeffreys’ entropy [22]) of two types of probability distributions can be defined to characterize the difference between the two types of distributions.

(3) J(P,Q)=D(P,Q)+D(Q,P)

Because the calculation of the logarithm is complicated, for the convenience of calculation, the following Formula (4) is used as the approximate measure of J(P,Q).

(4) $$\begin{array}{c} U\left(P,Q\right)={∥P-Q∥}^{2}=\sum_{i=1}^{n}{\left(p_{i}-q_{i}\right)}^{2}\\ \;\;\left\{\begin{array}{c} p_{i}=\frac{1}{N_{1}}\sum_{k=1}^{N_{1}}{\left(s_{k_{i}}^{\left(1\right)}\right)}^{2}\\ q_{i}=\frac{1}{N_{2}}\sum_{k=1}^{N_{2}}{\left(s_{k_{i}}^{\left(2\right)}\right)}^{2} \end{array}\right. \end{array}$$

where $N_{1}$ and $N_{2}$ denote the number of samples of class 1 and class 2, respectively; *i* stands for feature number; $s_{k_{i}}^{\left(1\right)}$ denotes the *i*-th feature of *k*-th sample in class 1; $s_{k_{i}}^{\left(2\right)}$ represents the *i*-th feature of *k*-th sample in class 2. The above features must be normalized.

### 2.4. The Discriminant Algorithm for Distinguishing Ability of Multi-Features

Assume that ${\left\{q_{u}^{i}\right\}}_{i,\ldots ,n}$ is a sub-template described by the *i*-th feature in the target area. The similarity value between the positive example and the target is $\rho_{m}^{i,pos}$, which represents the similarity value of the *m*-th positive example of the *i*-th feature point template, where m∈M. The similarity value between the negative example and the target is $\rho_{n}^{i,neg}$, which denotes the similarity value of the *m*-th negative example of the *i*-th feature point template, where n∈N. According to the problem description and the calculation method of discriminant entropy described above, the separability metric $J^{i}$ of each feature sub-template after normalization can be designed as:

(5) $$\begin{array}{c} J^{i}=\sum_{i=1}^{k}{\left({\bar{\rho }}^{i,pos}-{\bar{\rho }}^{i,neg}\right)}^{2}\\ \;\left\{\begin{array}{c} {\bar{\rho }}^{i,pos}=\frac{1}{M}\sum_{k=1}^{M}{\left(\rho_{k}^{i,pos}\right)}^{2}\\ {\bar{\rho }}^{i,neg}=\frac{1}{N}\sum_{k=1}^{N}{\left(\rho_{k}^{i,neg}\right)}^{2} \end{array}\right. \end{array}$$

where *k* denotes feature vector dimension of a sub-template; $\rho_{k}^{i,pos}$ denotes the similarity between the *k*-th positive example of the *i*-th feature sub-template and the target; $\rho_{k}^{i,neg}$ represents the similarity between the *k*-th negative example of the *i*-th feature sub-template and the target; ${\bar{\rho }}^{i,pos}$ denotes the mean squared of similarity of the positive example of the *i*-th feature sub-template; ${\bar{\rho }}^{i,neg}$ denotes the mean squared of similarity of the negative example of the *i*-th feature sub-template. All features are normalized.

According to Formula (5), $J^{i}$ measures the separability ability of the *i*-th feature sub-template. When $J^{i}$ is larger, the separability of the feature template is better. Therefore, we can find the best sub-template by calculating $\max(J^{i})$. According to the Winner-Takes-All criterion, when the sub-template of a feature has the best distinguishing ability, the next frame will use this feature to track. By calculating the value of this judge criterion, it can adaptively select the best feature, thereby achieving robust tracking in transform scenarios.

### 2.5. Selectively Update for Sub-Template

In the object tracking process, especially in the long-term object tracking, the update of template is a necessary step. Through the update of template, the tracking algorithm can better adapt to the changes of the environment and the object itself. Through the target update, the target template can be adjusted to achieve long-term tracking. However, there is a problem in the current target update: the drift of the template. In the paper [25], the target template uses the following two methods:

1. Adaptively update target size based on the characteristics of the maximum posterior probability similarity criterion;
2. Two thresholds are set to control the number and degree of template updating.

The above methods can be applied to a single feature target template or a multi-feature template in a high dimensional space. For the tracking algorithm based on adaptive feature selection in this paper, the complementarity between target feature sub-templates can be used to further reduce template drift. For example, tracking objects with color and texture features. If the object enters a bright environment from a dark environment, the color feature will change significantly due to illumination. The nature of the texture feature determines that it will not be affected by illumination. At this time, under the tracking framework based on adaptive feature selection, the texture feature will be used for image tracking during the environment switching, and the color feature will be used for tracking in the stable lighting environment.

Therefore, this paper proposes a method for selective updating of sub-template: in each frame, the tracking stable features are not updated, only the sub-templates with poor separability are updated. With this updated strategy, we can prevent the target template from introducing background information due to incorrect updates, thereby gaining adaptability to changes in the scene or object. The algorithm first sorts the feature sub-templates according to the discriminant entropy value; secondly, sets an update threshold λ, the feature sub-templates larger than this threshold are not updated, the feature sub-templates smaller than the threshold are updated.

In the specific algorithm, the two target update methods in paper [25] can be used to enhance the validity of the template update. In the tracking algorithm, the first method described above is used to adaptively adjust the target size. When determining the updating feature sub-template, a second method is used to control the speed of the update and the degree of the update. Through the above three methods, the algorithm can be adaptive to the target change.

### 2.6. Steps of Adaptive Feature Selection Algorithm

The similarity of the positive example, negative example, and target area of each feature sub-template can be calculated by simple linear summation. In this way, more positive and negative examples can be selected to better determine the distinguishing ability of the feature sub-template.

The process of adaptive selection algorithm based on color and texture features is as follows. First, initialize the various parameters of the algorithm. In the first frame, the color feature of target is described as $q_{u}^{1}$, and the texture feature is described as $q_{u}^{2}$. $y_{i}$ represents the target center of the initial position. ${\left\{x_{i}\right\}}_{i-1,\ldots ,m}$ denote the positions of each pixel in searching area in the first frame. Since there is no prior information in the first frame, either color or texture feature can be selected to start tracking. We select the color feature to start the tracking of the first frame.

1. Set the target position $y_{i}$ of the last frame as initial position. Calculate the color feature description $s_{u}^{1}$ and texture feature description $s_{u}^{2}$ in searching area centered on the initial position;
2. According to the formula $g\left(x_{i}\right)=\frac{q_{u}\left(x_{i}\right)}{s_{u}\left(x_{i}\right)}$, calculate the similarity contribution $g^{1}\left(x_{i}\right)$ and $g^{2}\left(x_{i}\right)$ of each pixel in the searching area in current frame. According to the separability result of the previous frame, the similarity contribution value of the corresponding feature is selected for tracking;
3. Initialize *k* = 0 which represents the number of initial iterations;
4. Calculate the position of the center to be matched of the next iteration by Formula (6). At the same time, set k=k+1.
  (6) $$y_{i+1}=\frac{\sum_{x_{i}\in A_{y_{i}}}x_{i}g\left(x_{i}\right)}{\sum_{x_{i}\in A_{y_{i}}}g\left(x_{i}\right)}$$
  where ${\left\{x_{i}\right\}}_{i-1,\cdots ,m}$ is the position of each pixel in the searching area; $y_{i}$ is the center of the area; $g\left(x_{i}\right)$ is the similarity contribution value of each pixel in the searching area; $A_{y_{i}}$ is the set of pixels representing the search area centered at $y_{i}$; $y_{i+1}$ is the target position of the next frame.
5. Repeat step 4 until $∥y_{i+1}-y_{i}∥<\varepsilon \;\mathrm{or}\;k\geq N$;
6. Calculate the distinguishing abilities of color and texture features in current scene by Formula (5);
7. According to Formula (7) [25], calculate whether the target size changes adaptively;
  (7) $$\omega \left(k+1\right)=\left\{\begin{array}{cc} \omega (k+2)+2, & \;\mathrm{if}\;{\bar{\phi }}_{-1}>0.8\;\mathrm{and}\;{\bar{\phi }}_{0}>0.75\;\mathrm{and}\;{\bar{\phi }}_{1}>0.6\\ \omega (k-2)-2, & \;\mathrm{if}\;{\bar{\phi }}_{0}<0.6\;\mathrm{and}\;{\bar{\phi }}_{1}<0.3\\ \omega (k), & \;\mathrm{otherwise}\; \end{array}\right.$$
  where *k* is the number of frames of the image; ω(k) represents the size of the target template in the *k*-th frame; ${\bar{\phi }}_{i}\left(i=1,\ldots ,a\right)$ is the average of the posterior probability measure (PPM) of each pixel; *a* is the comparison step of scale adaptation and is set to 1 without losing the generality.
8. According to Formula (8) [25], determine whether to update the target.
  (8) $$\bar{\phi }\left(p,q\right)\geq \delta$$
  where *p* represents the current frame template; *q* denotes the target template; ϕ(p,q) is the similarity of the posterior probability measure for the current frame and the target template; δ is the threshold parameter. If the sub-template needs to be updated, update the vector of the target feature sub-template according to Formula (9) [25]:
  (9) $$q^{\prime }=\gamma p+\left(1-\gamma \right)q$$
  where γ is the updated weighted value; *p* is the target feature vector of the current frame; *q* is the feature vector of the target template; $q^{\prime }$ is the updated target template feature vector;
9. Import the next picture in the sequence and jump to step 1.

During the iterative process of the entire algorithm, the characterization of the search area is calculated only once. In the actual algorithm process, only step 4 and step 5 are iterated.

## 3. Experiments

In this section, we first introduce the experimental datasets and evaluation metrics. Then, we describe the process and details of the experiment. The results of ablation and contrast experiment are shown in the third part. Finally, we analyze the process and speed of the experiment.

### 3.1. Experimental Dataset and Evaluation Metrics

**Figure 3:**
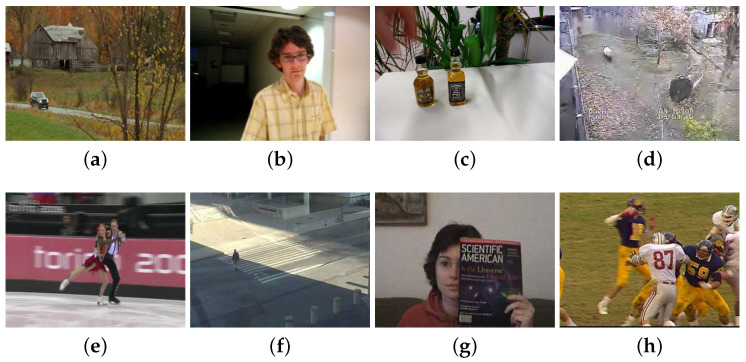
Eight test sequences used in experiment. (**a**) CarScale; (**b**) David; (**c**) Liquor; (**d**) Panda; (**e**) Skating2; (**f**) Crossing; (**g**) FaceOcc1; (**h**) Football1. These eight sequences possess the nine attributes described in the VTB dataset (SV, OCC, FM, etc.). For visual effects, some sequences have been stretched to achieve the same size.

In the experiment, we select eight video sequences from the Visual Tracker Benchmark (VTB) dataset [9] (http://cvlab.hanyang.ac.kr/tracker_benchmark/datasets.html, accessed on 7 June 2021), which is shown in Figure 3. These sequences have attributes such as scale variation, occlusion, deformation, and out-of-plane rotation, which are all representative in object tracking tasks.

To illustrate the effectiveness of this algorithm, we adopt two evaluation metrics to measure the results of the experiment in this paper. The former is the center location error (CLE) [26,27], which calculates the Euclidean distance of the center of the tracking box. It defined as:

(10) $$\mathrm{CLE}=\sqrt{{\left(x_{1}-x_{2}\right)}^{2}+{\left(y_{1}-y_{2}\right)}^{2}}$$

where $\left(x_{1},y_{1}\right)$ represents the center position of the ground truth label in a certain frame. $\left(x_{2},y_{2}\right)$ represents the center position of the tracking result label in a certain frame.

Another evaluation metric is the success rate (SR), which involves a concept commonly used in object tracking—Intersection over Union (IoU). It calculates the overlap ratio between the generated candidate box and the ground truth box. The formula of IoU is as follows:

(11) $$IoU=\frac{area(C)\cap area(G)}{area(C)\cup area(G)}$$

where area(C) represents the area of the generated candidate box, area(G) denotes the area of the ground truth box. The ideal situation is complete overlap, which means IoU equals 1. The threshold is set to 0.5. It is considered to have successfully tracked the object when the IoU of a frame reaches 0.5 or more. The SR is equal to the number of frames successfully tracked divided by the number of all frames.

### 3.2. Implementation Description

Our proposed SAFS algorithm can add multiple features for tracking. The color and texture features of the target are used to design the corresponding tracking algorithm. Readers can also add features such as gradients and edges to the algorithm.

Color information is an important global feature that is robust to target rotation, partial occlusion, and non-rigid transformation when color feature histograms are used to represent color information. In this algorithm, the feature histogram of the RGB color space is used to make the sub-template of the target color feature, and the RGB feature can be quantized into a feature vector of 8×8×8=512 dimensions.

In this paper, texture feature is selected as another feature sub-template, which is also a global feature. The original LBP feature operator [25] is chosen to describe the shape information of the object. For the original LBP feature operator with the radius of 1 and sampling points of 8, the 58-dimensional feature vector is obtained by rotation and equivalent transformation. The statistical histogram of LBP feature is also used to describe the texture feature sub-template. In the experiment, the tracking feature of the first frame selects the color feature. Table 1 lists the environment information used in our experiment.

### 3.3. Comparison Experiment

Our algorithm can adaptively select features between color and texture. In order to show the advantages of our self-adaptive algorithm, we select two algorithms that track only one feature and use them as baselines for comparison. We compare SAFS algorithm with the maximum posterior probability iterative tracking algorithm using texture feature alone (LBP&PPM) [21] and color feature alone (RGB&PPM) [21]. In addition, considering that SAFS is not based on deep learning, we should compare it with some classic algorithms. Therefore, we add three state-of-the-art traditional algorithms in the comparison, which are the mean-shift algorithm using the joint color-texture histogram (LBPT) [28], discriminative scale space tracking (DSST) [13] and high-speed tracking with kernelized correlation filters (KCF) [29]. All the algorithms are designed to track one object in our experiments.

From Table 2, we can easily see that our SAFS has the highest average SR. The DSST [13] gets 100 in “David” sequence, the RGB&PPM [21] gets 95 in “Skating2” sequence and 100 in “Crossing” sequences, which obtain better SR than SAFS. Meanwhile, SAFS achieves the best results in five sequences.

**Figure 4:**
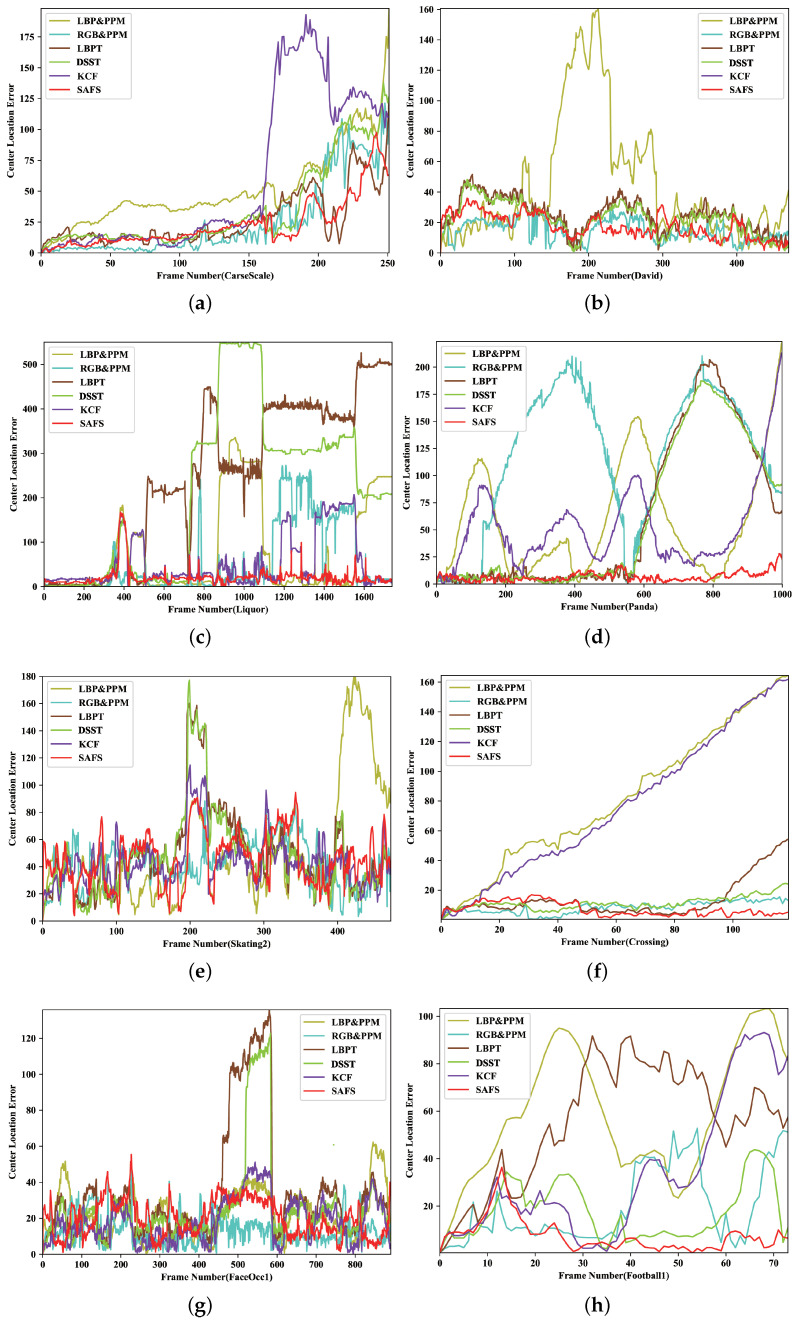
The experimental results of comparing the CLE (in pixel) frame by frame in eight sequences. Our SAFS algorithm can track the object robustly and accurately. (**a**) CarScale; (**b**) David; (**c**) Liquor; (**d**) Panda; (**e**) Skating2; (**f**) Crossing; (**g**) FaceOcc1; (**h**) Football1.

Table 3 lists the center location errors of 8 sequences using 6 methods, respectively. Our SAFS algorithm achieves the lowest average center location error which is 18 pixels. In addition, we display the results of the center-pixel location error frame by frame for eight sequences, as shown in Figure 4. The red line representing SAFS is always at the bottom of the figure, which means that its average CLE is minimal. Experimental results show that the algorithm can achieve robust tracking of the object when the object or scene changes. It should be pointed out that in the process of updating the target template, template drift still exists, which affects the tracking effect to a certain extent.

### 3.4. Tracking Process Analysis

**Figure 5:**
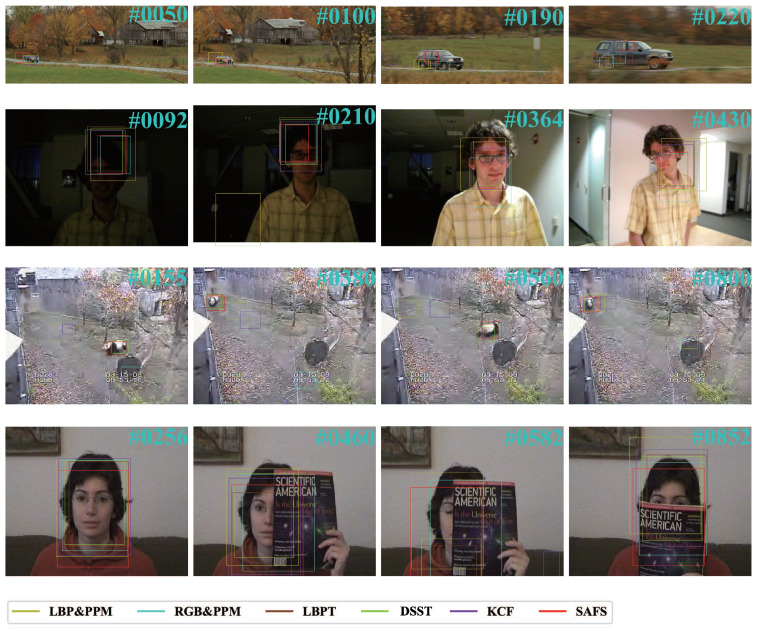
Experiment results of some fundamental frames of six algorithms on four sequences (CarScale, David, Panda, FaccOcc1).

To illustrate the difference of the algorithm, we analyze the tracking process of four sequences. Figure 5 lists the tracking conditions of the four fundamental frames in each sequence. “CarScale” is a sequence with scale variation, occlusion, and fast motion. “David” is a sequence with illumination variation, deformation, and motion blur. “Panda” is a sequence with scale variation, occlusion, and low resolution. “FaceOcc1” is a sequence with occlusion. The process analysis of the experimental results is as follows:

(1) CarScale: The most significant attribute of this sequence is scale variation. The scale of the car is increasing as it comes fast from afar. In the first 155 frames, all algorithms can track the object correctly. Starting from frame 165, KCF gradually loses the target and re-tracks the target at frame 201 the end because of the occlusion of the tree during these frames, which means KCF cannot handle occlusion issues well. From the overall point of view, LBPT and SAFS show better performance. Although most of the algorithms can track the target well, the tracking boxes cannot increase with the scale of the target. This is a point that our algorithm needs to pay attention to and improve.
(2) David: At first, the environment is dark. LBPT, DSST, KCF and SAFS get better results. From frame 162, LBP&PPM loses the object because the light is too dim, resulting in sparse texture feature. RGB&PPM loses the target between frame 451 and frame 536 because of the confusing effect of the poster on the wall. During the tracking process, although the LBPT has tracked the object, some offset has occurred. The reason for the offset is that when the color sub-template was updated in the previous tracking process, a template drift occurred, which resulted in a matching shift. The performance of LBPT shows that the combined feature of color and texture can simultaneously show the characteristics of different features in the tracking, but this type of feature will interfere with each other, resulting in inaccurate positioning of the target. DSST, KCF and SAFS can always track the object well.
(3) Panda: This is a long sequence with 1000 frames. LBP&PPM loses the target since frame 51 and RGP&PPM loses the target since frame 138. This indicates that a single feature cannot track the target well. LBPT and DSST lose the target when the panda passes the brand for the second time. KCF losses the target several times due to the occlusion of trees or interference of similar objects. Our SAFS algorithm is very robust, so it can track the target very well throughout the process.
(4) FaceOcc1: This is a relatively simple target tracking sequence because it only has one attribute—occlusion. From frame 523, LBPT and DSST lose the tracking of the face, which indicate that they cannot deal with the object occlusion well. Other algorithms show good performance in this sequence.

### 3.5. Speed Analysis

Table 4 lists the tracking frames per second (fps) and average fps of the six algorithms in this experiment. LBP&PPM [21] and RGB&PPM [21] both get high average fps in that they all use a single feature. The speeds of LBPT [28] and KCF [29] are 425 fps and 314 fps, respectively, which are slower than ours. DSST [13] is a low-speed algorithm, which gets the slowest average speed 85. Our SAFS gets a medium speed (467). Its main time-consuming steps are in the description of features and the selection of sub-templates. Therefore, we can consider appropriately reducing the calculation of positive and negative examples and narrowing the search area to increase the tracking speed. Furthermore, if more features are introduced for object tracking, the speed of the algorithm will be closely related to the types of features and the dimensions of feature vectors.

## 4. Conclusions

In this paper, we propose an object tracking method based on a new self-adaptive feature selection algorithm, called SAFS. This method can overcome the challenge of robustness by selecting the most suitable feature, which is a significant problem in the image domain. Furthermore, our proposed method can achieve faster inference speed, which is the inevitable factor in edge application.

To evaluate the performance of the SAFS, we test it on eight different video sequences from comprehensive perspectives. Our proposed method can obtain a higher success rate and shrink the center location errors in various degrees, and even outperform 90% compared to other baselines in some specific scenes. Future work will be dedicated to decreasing the complexity of the algorithm and further improving the performance.

## Figures and Tables

**Table 1:** The experimental environment information.

System	CPU	Frequency	RAM	Software
Windows10	Intel (R) Core (TM) i7-7700	3.60 GHz	16.0	MATLAB R2019b

**Table 2:** The SR (%) of the SAFS method compared with other five methods.

Video Sequence	LBP&PPM [21]	RGB&PPM [21]	LBPT [28]	DSST [13]	KCF [29]	SAFS
CarScale	89	100	100	85	88	100
David	71	99	96	100	99	99
Liquor	68	75	28	39	75	98
Panda	27	16	58	57	18	100
Skating2	71	95	90	83	92	91
Crossing	11	100	80	97	12	96
FaceOcc1	100	100	96	91	100	100
Football1	24	76	24	90	61	95
Average SR	57.6	82.6	71.5	80.3	92.5	97.3

**Table 3:** The CLE (pixels) of the SAFS method compared with other five methods.

Video Sequence	LBP&PPM [21]	RGB&PPM [21]	LBPT [28]	DSST [13]	KCF [29]	SAFS
CarScale	51	25	27	33	59	23
David	58	15	25	21	18	7
Liquor	76	54	251	205	53	21
Panda	27	117	60	57	52	7
Skating2	52	42	48	45	43	45
Crossing	81	8	14	9	76	8
FaceOcc1	22	13	34	24	19	20
Football1	59	19	55	17	31	8
Average CLE	53	37	64	53	44	18

**Table 4:** Speed comparison of six algorithms.

Sequence	LBP&PPM [21]	RGB&PPM [21]	LBPT [28]	DSST [13]	KCF [29]	SAFS
CarScale	1139	2357	628	132	464	732
David	297	734	123	27	124	259
Liquor	120	234	97	89	312	60
Panda	1702	3220	1294	203	721	1061
Skating2	99	290	48	11	41	63
Crossing	1251	1561	793	128	523	1029
FaceOcc1	136	197	94	9	37	50
Football1	897	1147	324	78	287	486
Average FPS	705	1217	425	85	314	467
